# Cultured skin microbiota attracts malaria mosquitoes

**DOI:** 10.1186/1475-2875-8-302

**Published:** 2009-12-17

**Authors:** Niels O Verhulst, Hans Beijleveld, Bart GJ Knols, Willem Takken, Gosse Schraa, Harro J Bouwmeester, Renate C Smallegange

**Affiliations:** 1 Laboratory of Entomology, Wageningen University and Research Centre, P.O. Box 8031, 6700 EH Wageningen, the Netherlands; 2 Plant Research International, Wageningen University and Research Centre, P.O. Box 16, 6700 AA Wageningen, the Netherlands; 3 Laboratory of Microbiology, Wageningen University and Research Centre, P.O. Box 8033, 6700 EJ Wageningen, the Netherlands; 4 Laboratory of Plant Physiology, Wageningen University and Research Centre, Arboretumlaan 4, 6703 BD Wageningen, the Netherlands; 5 NIZO food research B.V., Afd. Flavour, Kernhemseweg 2, 6718 ZB, Ede, the Netherlands; 6 Division of Infectious Diseases, Tropical Medicine & AIDS, Academic Medical Center, F4-217 Meibergdreef 9, 1105 AZ, Amsterdam, the Netherlands

## Abstract

**Background:**

Host-seeking of the African malaria mosquito, *Anopheles gambiae sensu stricto*, is guided by human odours. The precise nature of the odours, and the composition of attractive blends of volatiles, remains largely unknown. Skin microbiota plays an important role in the production of human body odours. It is hypothesized that host attractiveness and selection of *An. gambiae *is affected by the species composition, density, and metabolic activity of the skin microbiota. A study is presented in which the production and constituency of volatile organic compounds (VOCs) by human skin microbiota is examined and the behavioural responses of *An. gambiae *to VOCs from skin microbiota are investigated.

**Methods:**

Blood agar plates incubated with skin microbiota from human feet or with a reference strain of *Staphylococcus epidermidis *were tested for their attractiveness to *An. gambiae *in olfactometer bioassays and indoor trapping experiments. Entrained air collected from blood agar plates incubated with natural skin microbiota or with *S. epidermidis *were analysed using GC-MS. A synthetic blend of the compounds identified was tested for its attractiveness to *An. gambiae*. Behavioural data were analysed by a χ^2^-test and GLM. GC-MS results were analysed by fitting an exponential regression line to test the effect of the concentration of bacteria.

**Results:**

More *An. gambiae *were caught with blood agar plates incubated with skin bacteria than with sterile blood agar plates, with a significant effect of incubation time and dilution of the skin microbiota. When bacteria from the feet of four other volunteers were tested, similar effects were found. Fourteen putative attractants were found in the headspace of the skin bacteria. A synthetic blend of 10 of these was attractive to *An. gambiae*.

**Conclusions:**

The discovery that volatiles produced by human skin microorganisms *in vitro *mediate *An. gambiae *host-seeking behaviour creates new opportunities for the development of odour-baited trapping systems. Additionally, identification of bacterial volatiles provides a new method to develop synthetic blends, attractive to *An. gambiae *and possibly other anthropophilic disease vectors.

## Background

The African malaria mosquito *Anopheles gambiae sensu stricto *(hereafter referred to as *An. gambiae*) preferably feeds on human beings inside houses and is therefore one of the most effective vectors of *Plasmodium *malaria parasites [1]. Although visual and physical cues play a role in the host-seeking behaviour of *An. gambiae*, host-seeking is mainly accomplished by odour-mediated anemotaxis in which volatile organic compounds (VOCs) of human origin provide essential cues [1]. Humans are differentially attractive to mosquitoes because of the odours they emit [2-4]. The skin microbiota plays an important role in the production of human body odours [5] and without bacteria human sweat is odourless [6]. Many volatile compounds seem to be widespread among bacteria, although others are strain-specific. Some strains can produce up to 60 different volatile compounds [7]. Differences in foot odour production can be explained by micro-floral differences between humans [8] and a recent study by Xu *et al *[9] provides more evidence that there is a connection between the microbial composition on human skin and chemical signature of humans. If host-selection by *An. gambiae *is based on the species composition, metabolic activity and/or density of the skin microbiota, then this will bear a direct impact on the number of bites received per person and the resulting risk of infection [1]. The non-random nature of host selection remains poorly understood, yet has an important impact on exposure to disease [10].

A study on Limburger cheese volatiles revealed the putative role of bacteria in mosquito olfaction [11,12]. Knols *et al *[11] suggested that bacteria involved in the ripening of Limburger cheese may have originated from human skin and hence that these bacteria are responsible for the production of 'human-specific' VOCs that mediate the host-seeking process of malaria mosquitoes. Washing the feet with a bactericidal soap significantly altered the selection of biting sites of *An. gambiae *on a motionless naked volunteer [13]. In addition, human eccrine sweat is attractive to *An. gambiae*, but only after incubation for one or two days [14]. Microorganisms on the skin are responsible for the conversion of fresh sweat into sweat attractive to *An. gambiae *[6,14].

The human odour profile consists of more than 350 compounds [15,16]. It was examined whether human skin microbiota produces attractive VOCs (kairomones) [17] for *An. gambiae *when cultured *in vitro *and whether analysis of entrained odours collected from these microorganisms can lead to a synthetic blend attractive to *An. gambiae*. In this paper an attractant is defined as a compound or blend of compounds, which causes insects to make oriented movements towards its source [18].

## Methods

### Insects

The *An. gambiae *s.s. culture originated from Suakoko, Liberia (courtesy Prof. M. Coluzzi). Mosquitoes have been cultured in the laboratory since 1988 and received blood meals from a human arm twice a week. Adults were maintained in 30-cm cubic gauze-covered cages in a climate-controlled chamber (27 ± 1°C, 80 ± 5% RH, LD 12:12). They had access to a 6% (w/v) glucose solution on filter paper. Eggs were laid on wet filter paper and placed in tap water in plastic trays and fed daily with Tetramin^® ^baby fish food (Melle, Germany). Pupae were collected daily and placed in 30-cm cubic cages for emergence.

### Skin microbiota sampling

Skin microbiota samples were taken from a human foot, because there is evidence that this body part produces VOCs that influence the selection of biting sites by *An. gambiae *[19]. Each volunteer (all healthy males, Caucasian, aged 23, 25, 28, 29, and 31 years) was asked not to shower, drink alcohol and eat spicy food 24 hours before the experiment and not to use soap during the last shower. Volunteers were provided a nylon sock, which had to be worn 24 hours before the experiment. Samples were taken from the foot of each volunteer by using a sampling ring and washing buffer as described by Taylor *et al *[20]. A sterile Teflon sampling ring (internal diameter 2.9 cm) was placed in the centre of the underside of the foot, and 0.75 mL of full-strength wash fluid (75 mM sodium phosphate buffer (pH 7.9) + 0.1% (v/v) Triton X-100, Merck, The Netherlands) was added. The surface of the skin, within the ring, was gently scrubbed with a sterile glass stick for 1 min and the fluid was pipetted in a 2 ml sample tube (Eppendorf^®^). Immediately thereafter the process was repeated at the same site, and the two samples were pooled and diluted 5× in half strength wash fluid [20]. Diluted microbiota samples (100 μl) were spread on Colombia (sheep) blood agar plates (Tritium, The Netherlands; http://www.tritium-microbiologie.nl/) before use in the behavioural experiments.

The number of colony-forming units (cfu) in the samples taken from the human feet was determined using selective plates for the five microbiota genera most abundant on human skin. According to the method described by Taylor *et al *[20], media were selective for staphylococci, aerobic corynebacteria, micrococci, propionibacteria and *Pityrosporum *species (Tritium, The Netherlands).

**Figure 1:**
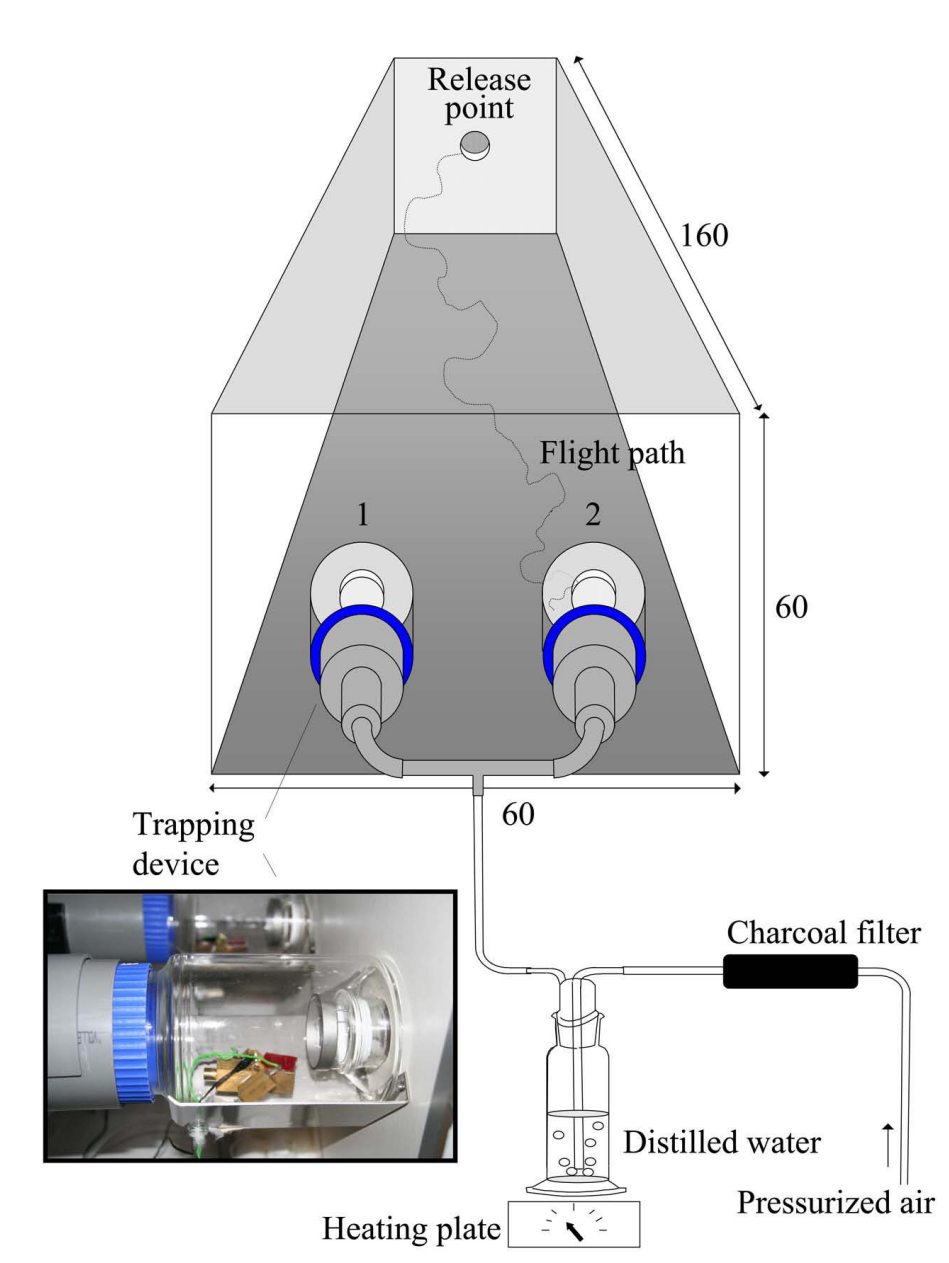
**Dual-choice olfactometer with two CO_2 _measurement positions (1, 2) **[14,21]. The photograph shows a trapping device and an excised block of blood agar with microbiota on a heated brass block, regulated at 34 ± 0.2°C by a universal thermostat with external sensor. All dimensions are in cm.

Olfactometer bioassaysA dual-port olfactometer (Figure 1) [14,21] was used to evaluate host-seeking responses of female mosquitoes to VOCs produced by microbiota from the human skin. Pressurized air was charcoal-filtered, humidified, and passed through two glass mosquito trapping devices, which were linked to both ports (diameter 5 cm, 30 cm apart) of the olfactometer. The air entered the flight chamber (1.60 × 0.60 × 0.60 m) with a speed of 22 ± 1 cm/s, temperature of 28.3 ± 0.5°C, and relative humidity above 80%. The experimental room was maintained at a temperature of 26.7 ± 0.8°C and a relative humidity of 64.5 ± 3.5%.

Experiments were prepared and performed according to the methods described by Smallegange *et al *[22]. For each test 30 (mated) female mosquitoes of 5-8 d old, which had never received a blood meal, were selected 14-18 h before the experiment and placed in a cylindrical release cage (d = 8, h = 10 cm) with access to tap water from damp cotton wool. The experiments were performed during the last 4 h of the scotophase, when *An. gambiae *females are known to be highly responsive to host odours [23,24]. In each trial, test odours were released in the air stream before a group of mosquitoes was set free from a cage which was placed at the downwind end of the flight chamber, 1.60 m from the two ports. Mosquitoes were left in the flight chamber for 15 min. Specimens that entered each of the two trapping devices were counted at the end of the experiments. Mosquitoes remaining in the flight chamber were removed with a vacuum cleaner. Each trial started with a fresh batch of mosquitoes, clean trapping devices, and new stimuli. A randomized complete block design was used which included five test treatments and one control treatment (so six treatments total) over six days. For each treatment, 30 female mosquitoes were released in the olfactometer. The sequence of test odours was randomized on the same day and between days. Test stimuli were alternated between right and left ports in different replicates to rule out any positional effects. Surgical gloves were worn by the researcher at all times to avoid contamination of equipment with human volatiles.

Excised blocks of blood agar (1.5 × 1.5 × 0.3 cm) with or without microbiota were placed on a glass slide (1.5 × 1.5 cm) and then heated on a brass block (Figure 1). One blood agar block was placed in each trapping device. The temperature of each block (34.0 ± 0.2°C) was regulated with a universal thermostat with external sensor (UT 100, Conrad electronic, The Netherlands).

In the first series of experiments diluted microbiota samples (100 μl) of one of the volunteers (28 years old) were spread on Colombia (sheep) blood agar plates (Tritium, The Netherlands) and incubated for 0, 6, 12, 24, 36, 72 hours at skin temperature (34°C) [25] before testing. Small blocks (1.5 × 1.5 × 0.3 cm) of blood agar incubated with skin microbiota were tested against small blocks of sterile blood agar.

In the second series of experiments all samples, originating from one of the volunteers (28 years old), were incubated for 12 h, after it had been decimally diluted (1:1; 1:10; 1:100; 1:1,000 or 1:10,000). Sterile control blood agar plates were incubated together with the plates with the skin microbiota. Excised blocks of blood agar incubated with skin microbiota were tested against blocks of sterile blood agar.

For a third series of experiments a bacterial sample was taken as described above from five volunteers (male, Caucasian) to test whether the main result obtained by using bacteria of the volunteer in the first series of experiments could be repeated with samples from other volunteers. The volunteer from the first series of experiments was one of the five volunteers in this experiment (# 4). Bacterial samples of each volunteer were diluted to a concentration of 2.63·10^2 ^cfu per cm^2 ^(similar as found in the first series of experiments), spread on blood agar plates and incubated for 12 hours (based on the result of the first series of experiments). Blocks of blood agar with microbiota from the volunteers were tested against a control of sterile blood agar in the olfactometer.

To verify that *An. gambiae *is attracted to VOCs released by microorganisms common on human skin [26], an olfactometer experiment was conducted with a reference strain of *Staphylococcus epidermidis *(DSMZ 11047). Small blocks (1.5 × 1.5 × 0.3 cm) of blood agar grown with *S. epidermidis *at a concentration of 2.63·10^2 ^cfu per cm^2 ^for 12 hours were tested against a control of sterile blood agar in the olfactometer.

In the first series a negative control of blood agar with microbiota that was not incubated was randomized with the treatments. In the second and third series two excised blocks of blood agar (1.5 × 1.5 × 0.3 cm) without microorganisms were tested against each other and randomized with the treatments as a control.

Experiments in which only clean moist air was released from both ports of the olfactometer were conducted to test the symmetry of the trapping system. Six tests were performed on one day which showed that the system was symmetrical (χ^2^-test, d.f. = 1, P = 1.00, trap entry response 14.5%).

### Experimental room trapping

**Figure 2:**
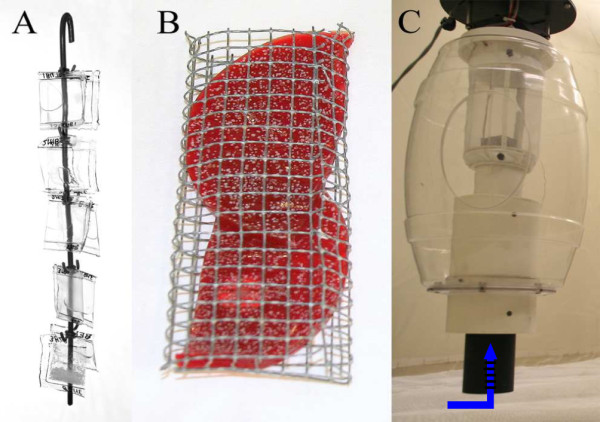
**Odour release methods in MM-X traps**. (A) Hook with 10 LDPE sachets for release in MM-X traps; (B) metal holder with blood agar with bacteria (11.5 × 5 × 1 cm) for adjustment in inner tube; (C) MM-X trap, arrow indicates the position of LDPE sachets (A) and agar samples (B).

Two Mosquito Magnet-X (MM-X) (American Biophysics Corp., USA) [27] traps were placed in a large netting cage of 233 × 250 × 330 cm (Howitec Netting BV, Bolsward, The Netherlands), inside a climate controlled room (T = 25 ± 0.5°C, RH = 72 ± 4%). The traps were placed at 2 m distance from each other. Blood agar plates with a mix of skin bacteria (2.63·10^2 ^cfu per cm^2^) were incubated for 12 hours at 34°C and tested against incubated sterile blood agar plates. Before the experiment, blood agar with or without skin microbiota was cut into two pieces, and placed inside a metal holder (11.5 × 5 × 1 cm). These holders (Figure 2) were then placed in the air outlet of a MM-X trap.

For each test 50 (mated) female mosquitoes, five to eight days old, which had never received a blood meal, were selected 14-18 h prior to the experiment and placed in a cylindrical release cage (d = 8, h = 17.5 cm) with access to tap water from damp cotton wool. The experiments were performed during the last 4 h of the scotophase. The mosquitoes were released from the release cage in the centre of the large cage. After 4 h, the MM-X traps were closed and transferred to a freezer to kill the mosquitoes. Experiments were repeated for six days, altering the side of each treatment every day. Surgical gloves were worn to avoid contamination of equipment with human volatiles.

On six mornings, experiments with unbaited traps in the MM-X setup were done to test the symmetry of the trapping system. Sterile blood agar was tested against sterile agar without blood to test the effect of the blood in the agar. As a control, sterile blood agar (1.5 × 1.5 × 0.3 cm) was tested against sterile blood agar.

A blend of ten volatile compounds originating from incubated skin bacterial samples and identified in the first part of the study by GC-MS (see below) (1-butanol; 2,3-butanedione; 2-methyl-1-butanol; 2-methylbutanal; 2-methylbutanoic acid; 3-hydroxy-2-butanone; 3-methyl-1-butanol; 3-methylbutanal; 3-methylbutanoic acid; benzeneethanol) were tested in the MM-X setup. Only compounds that were found to be significantly more abundant in the bacterial samples than in the control of sterile agar were tested (Table 1), except for 2-hydroxy-3-pentanone, which is not commercially available, and the three compounds that could not be identified.

**Table 1:** Compounds present in the odour blend which was tested in experimental room trapping experiment.

Compound (dilutions in H_2 _O)	Release rates μg/h	Supplier	Purity	LDPE thickness
1-butanol	168	Sigma	>99%	0.10 mm
2,3-butanedione (1:1000)	48	Fluka	>99%	0.10 mm
2-methyl-1-butanol	545	Sigma	>99%	0.03 mm
2-methylbutanal (1:1000)	31	Sigma	95%	0.10 mm
2-methylbutanoic acid (1:1000)	39	Sigma	98%	0.10 mm
3-hydroxy-2-butanone(solid)	32	Sigma	≥ 97%	0.03 mm
3-methyl-1-butanol	431	Fluka	≥ 99.8%	0.10 mm
3-methylbutanal (1:1000)	29	Fluka	≥ 98%	0.10 mm
3-methylbutanoic acid (1:1000)	34	Sigma	99%	0.10 mm
Benzeneethanol	261	Fluka	≥ 99%	0.05 mm
				
distilled water 0.10 mm	31			0.10 mm
distilled water 0.05 mm	131			0.05 mm
distilled water 0.03 mm	186			0.03 mm

Each compound was released from a separate LDPE sachet. The thickness of the sachets determined the release rates (μg/h), which was measured by weighting the LDPE sachets before and after the experiment.

One hundred μL of each compound (Fluka, Sigma, ≥ 95%; Table 1), either pure or diluted in distilled water, was dispensed from sealed sachets (25 × 25 mm) of Low Density PolyEthylene (LDPE; Audion Elektro, The Netherlands). The thickness of the polyethylene material was varied to adjust release rates for each compound [28] (Table 1). LDPE sachets were suspended from a hook and placed inside the black tube of the MM-X trap (Figure 2). The control consisted of an equal number of sachets with the same size and thickness as the sachets containing experimental compounds, but filled with distilled water only. Release rates (μg/h) were measured by weighing the sachets before and after the experiments [28].

### Volatile entrainment and GC-MS analysis

**Figure 3:**
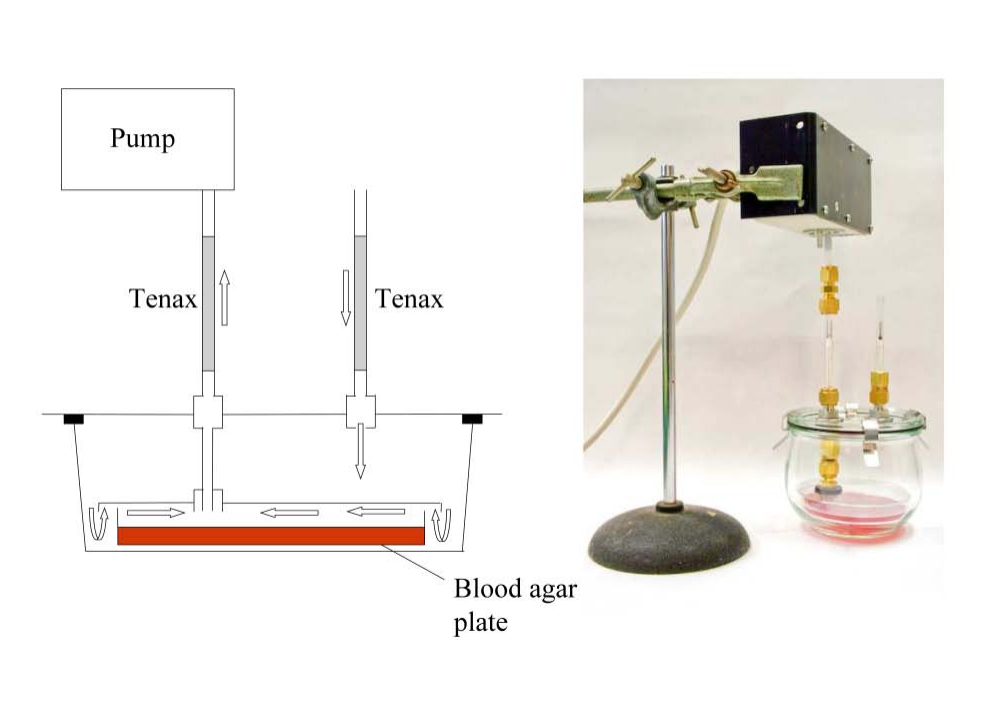
**Headspace sampling method of skin bacteria**. Schematic representation and photograph of the volatile sampling method used to collect the headspace of blood agar plates incubated with or without skin microbiota. Arrows indicate the direction of the airflow.

Volatiles were entrained using purge and trap (Figure 3) on Tenax-TA 20/35 (Alltech), from blood agar plates with different concentrations of human foot bacteria (n = 2 for each concentration). In addition, headspace samples of *S. epidermidis *on agar plates at a concentration of 2.63·10^2 ^cfu per cm^2 ^were taken (n = 4). Control samples consisted of sterile blood agar plates with wash buffer without bacteria added to it. Plates that had been incubated for 12 hours at 34°C were placed in a cuvette. To reduce background volatiles, air was sucked into the cuvette through a standard glass cartridge containing 100 mg Tenax-TA (Figure 3). Headspace volatiles were entrained at a flow rate of 100 ml/min for two hours on a second cartridge containing 100 mg Tenax-TA connected to the outlet of the cuvette. The whole setup was placed in an incubator at 34°C to allow growth of the bacteria at skin temperature. Tenax-TA cartridges were conditioned before the experiments by heating for 1 hour at 320°C under a flow of He (60 mL/min).

Samples were analysed by thermal desorption from the cartridge onto a cold trap and subsequent thermal desorption for introduction into the Trace GC Ultra coupled to a Trace DSQ (both from Thermo Scientific, The Netherlands). The thermal desorption programme consisted of a 3 min He dry purge and 1 min He prepurge both at 30°C. Cartridge desorption was performed at 250°C for 3 min and the volatiles were focused on a general purpose hydrophobic cold trap (Markes) at 0°C. Injection onto the analytical column was achieved by heating of the cold trap at the maximum speed (>60°C/s) to 250°C and splitting of the carrier gas (He) resulting in an injection of 1/6 of the total volatile amount. The transfer line between the cold trap and the GC was kept at 160°C.

A 30 m × 0.25 mm ID × 1.0 μm Rtx-5 MS with He carrier gas (1.0 mL/min) was used. The GC oven temperature programme was: 3 min at 45°C, ramping of 8°C/min to 280°C and a 2 minute hold at 280°C. The transfer line between the GC and MS was kept at 275°C. Mass-spectra were recorded by electron impact ionization at 70 eV, scanning in positive mode from 35-300 *m/z *with a scan speed of five scans/s and an ion source temperature of 250°C. The filament was switched off from 13.6-13.8 min because of a high background peak.

Peak analysis was performed using Xcalibur software and peak deconvolution by AMDIS, http://chemdata.nist.gov/mass-spc/amdis/. The obtained spectra were compared to the NIST-library. Calculated and reported retention indices and injection of authentic synthetic reference compounds (Table 1) provided additional information for identification.

### Carbon dioxide measurements

Carbon dioxide (CO_2_) is assumed to play an important role in mosquito host-seeking behaviour [29]. Therefore, CO_2 _levels in the olfactometer were measured on two days. Simultaneously, *An. gambiae *females were released to investigate whether their response to skin microbiota is (partly) due to CO_2 _emission from the skin microbiota. The concentration of CO_2 _inside the olfactometer was measured at two different positions (Figure 1) using a Xentra 4100 CO_2 _analyzer (Servomex, The Netherlands), at intervals of 3 min, according to the method described by Spitzen *et al *[30]. Carbon dioxide concentrations were measured over a range of 0 - 1030 ppm with an accuracy of 0.1 ppm. The data were downloaded to a PC using Das Wizard^© ^2.0 software (Measurement Computing Corporation, USA).

For this purpose, blood agar plates with a concentration of skin microorganisms of 2.63·10^2 ^cfu per cm^2^, incubated for 12 h at 34°C, were prepared, and blocks of 1.5 × 1.5 × 0.3 cm, heated on a brass block (34°C), were tested in the olfactometer.

### Statistics

For each two-choice test in the olfactometer and MM-X setup a χ^2^-test was used to analyze whether the total (i.e. sum of all replicates) number of mosquitoes that was trapped in the treatment trapping device and the total number that was trapped in the control trapping device differed from a 1:1 distribution (P < 0.05). A Generalized Linear Model (GLM, P < 0.05; Genstat for Windows, release 9.2) with binomial function, linked in logit, dispersion estimated, was used to investigate the effect of treatments on the trap entry response in the olfactometer experiments. The trap entry response is defined as the number of female mosquitoes caught in both trapping devices as the percentage of mosquitoes that flew out of the release cage [3].

Differences in CO_2 _concentrations at the different time intervals and between blood agar with or without skin microbiota were tested using a t-test for each time-interval (P < 0.05; Genstat for Windows, release 9.2).

The abundance of compounds in the chromatograms of the GC-MS analysis were fitted to an exponential regression line (Genstat for Windows, release 9.2) to test whether the concentration of microbiota present on blood agar plates had a significant effect on the abundance of each compound in the headspace samples of these plates (P < 0.01).

Differences between the abundance of compounds in the chromatograms of *S. epidermidis *and sterile blood agar plate headspace samples were tested using ANOVA. When a treatment effect was found (ANOVA, P < 0.05) a t-test was used to compare pairwise differences of the mean (Genstat for Windows, release 9.2).

## Results

### Olfactometer bioassays

**Figure 4:**
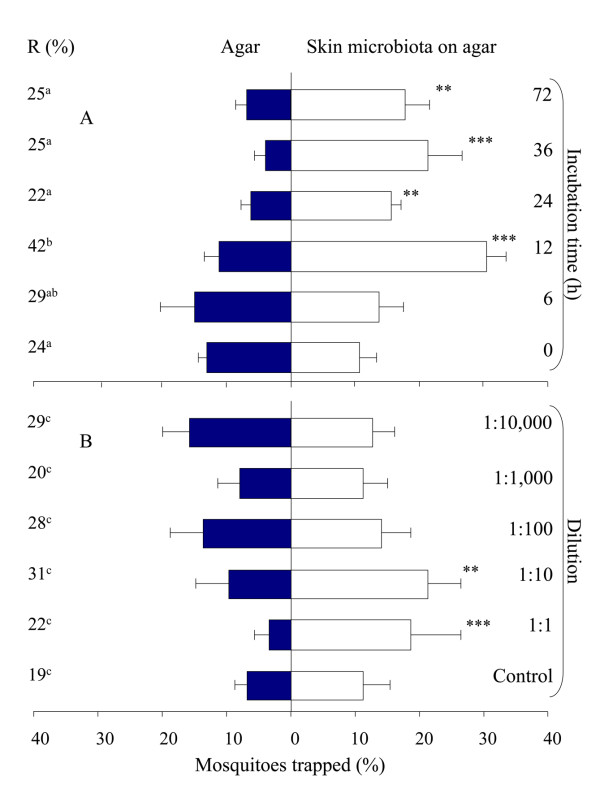
**Mean response of *An. gambiae *to skin microbiota in an olfactometer**. Microbiota were grown on blood agar and tested at different times of incubation (A) and in different dilutions of the most attractive treatment (12 h incubation) (B). Six times 30 mosquitoes were released per treatment. Error bars represent standard errors of the mean; ***: χ^2^-test P < 0.001; **: χ^2^-test P < 0.01. R = The trap entry response expressed as the number of female mosquitoes caught in both trapping devices divided by the number of mosquitoes that flew out of the release cage. Data followed by different letters differ significantly at P < 0.05 (GLM).

Traps with sterile blood agar caught significantly more mosquitoes than traps with clean moist air (χ^2^-test, d.f. = 1, P < 0.001). Blood agar on which skin microbiota were growing (initial concentration 2.63·10^2^cfu per cm^2^), however, caught significantly more mosquitoes than sterile blood agar after 12, 24, 36 and 72 h of incubation (Figure 4A; χ^2^-test, d.f. = 1, P < 0.01). The trap entry response, expressed as the number of female mosquitoes caught in both trapping devices divided by the number of mosquitoes that flew out of the release cage, was significantly higher during the tests with microbiota incubated for 12 h than with the other treatments (GLM, d.f. = 5, P < 0.05), except for the samples that had been incubated for 6 h (Figure 4A).

Based on colony counts on blood agar plates, the bacterial density on the sole of the volunteer's foot was estimated to be 1.90·10^5 ^cfu per cm^2^. Selective plates showed that staphylococci were most abundant (1.86·10^5 ^cfu per cm^2^); corynebacteria (5.22·10^4^cfu per cm^2^) and propionibacteria (4.54·10^3 ^cfu per cm^2^) were present in lower numbers. Micrococci and *Pityrosporum *were not found during any of the experiments.

In the second series of experiments the 1:10 dilution was chosen such that the concentration of bacteria before incubation was the same as in the first experiment (2.63·10^2 ^cfu per cm^2^), and higher and lower concentrations could be tested (1:1; 1:100; 1:1,000 and 1:10,000). The total bacterial density on the sole of the foot was estimated to be 1.14·10^7 ^cfu per cm^2^, the staphylococci density 9.27·10^6^, corynebacteria 2.16·10^6 ^and propionibacteria 5.73·10^5 ^cfu per cm^2^. Traps with blood agar with skin microbiota dilutions of 1:1 or 1:10 caught significantly more *An. gambiae *than traps with sterile blood agar (Figure 4B; χ^2^-test, d.f. = 1, P < 0.01). The trap entry response was not significantly different between treatments (Figure 4B; GLM, d.f. = 5, P > 0.05). The control experiments with sterile blood agar on both sides showed no positional bias (Figure 4B; χ^2^-test, d.f. = 1, P = 0.157).

In the third series of experiments the blood agar with bacteria from each volunteer (2.63·10^2 ^cfu per cm^2^, 12 h incubation) caught significantly more *An. gambiae *than the sterile blood agar (χ^2^-test, d.f. = 1, P < 0.05).

Carbon dioxide levels measured at the outlet of both ports of the olfactometer were equal at both ports of the olfactometer when incubated (12 h) blood agar with microbiota was tested against sterile blood agar (Table 2; t-test, d.f. = 1, P > 0.05).

**Table 2:** Mean carbon dioxide concentrations during behavioural experiments in the olfactometer.

Mean CO_2 _concentration (ppm) ± SE			
Time (min)	Sterile blood agar	Skin microbiota on blood agar	P-value
1-3	439.90 ± 1.63	441.18 ± 1.72	0.58
4-6	438.02 ± 1.58	438.59 ± 1.63	0.80
7-9	435.20 ± 1.52	435.60 ± 1.58	0.85
10-12	432.74 ± 1.48	434.09 ± 1.52	0.52
13-15	430.71 ± 1.43	432.17 ± 1.47	0.48

Carbon dioxide concentrations (ppm ± SE) were measured in front of the trapping device (Figure 1) with skin microbiota on blood agar and in front of the trapping device with sterile blood agar. Time indicates minutes after release of mosquitoes and start of each experiment.

### Volatile entrainment and GC-MS analysis

**Figure 5:**
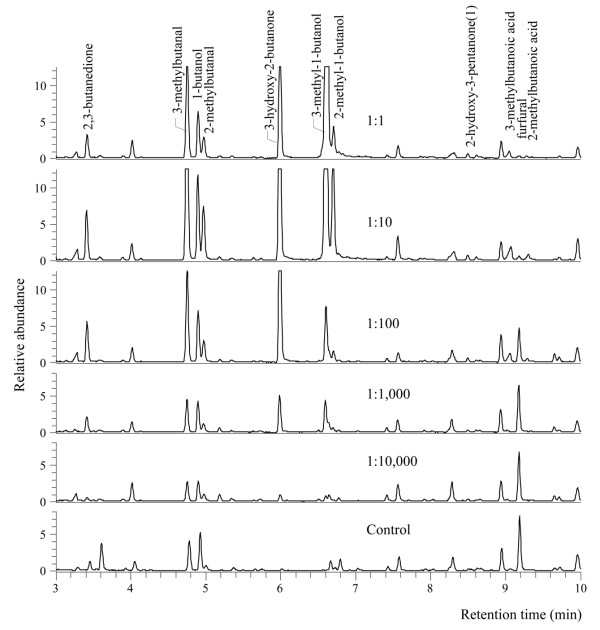
**GC-MS chromatogram of headspace samples of skin microbiota**. Part of GC-MS chromatograms of headspace samples of blood agar plates with different dilutions of skin microbiota and a blood agar plate without skin microbiota showing 11 compounds that were present in significantly higher or lower amounts in the control (Table 3). All compounds were identified on the basis of matching spectra (NIST library), and verified by injection of a standard (except 2-hydroxy-3-pentanone).

Regression analysis of the results of the volatile entrainment of headspace odours from the blood agar with diluted microbiota, originating from a human foot, and from the sterile (control) blood agar revealed 14 compounds that were more abundant when bacterial concentrations were higher (Table 3, Figure 5; exponential regression, d.f. = 7, P < 0.01). The 1:1 concentration was not included in the analysis because the abundance of compounds in the chromatograms showed a clear decline at this concentration (Figure 5), probably because of bacterial overgrowth on the plate.

**Table 3:** Bacterial headspace compounds.

Compounds found in lower amounts in diluted samples and control	RT (min)	R^2 ^(%)	P-value	Exponential regression parameters A + B*(R**X)			Studies on human odour
				A	B	R	
1-butanol^1^	4.89	83.2	< 0.001	2.21*10^7^	-1.79*10^7^	3.09*10^-16^	[16,41]
2,3-butanedione^1^	3.42	78.1	0.002	2.34*10^7^	-1.99*10^7^	3.77*10^-246^	
2-hydroxy-3-pentanone	8.49	94.1	< 0.001	1.39*10^6^	-1.02*10^6^	8.54*10^-26^	
2-methyl-1-butanol^1^	6.70	97.1	< 0.001	-4.93*10^6^	5.05*10^6^	2.89*10^4^	[16]
2-methylbutanal^1^	4.97	98.7	< 0.001	1.11*10^7^	-1.04*10^7^	1.32*10^-13^	[15,39]
2-methylbutanoic acid^1^	9.30	96.8	< 0.001	1.52*10^6^	-1.41*10^6^	8.56*10^-22^	[16]
3-hydroxy-2-butanone^1^	5.99	95.0	< 0.001	8.51*10^7^	-8.24*10^7^	2.31*10^-34^	[16]
3-methyl-1-butanol^1^	6.61	97.0	< 0.001	-5.12*10^7^	5.22*10^7^	4.25*10^3^	[16]
3-methylbutanal^1^	4.75	98.1	< 0.001	2.74*10^7^	-2.51*10^7^	7.89*10^-15^	[39]
3-methylbutanoic acid^1^	9.07	92.9	< 0.001	5.42*10^6^	-4.69*10^6^	6.63*10^-28^	[16,41]
benzeneethanol^1^	15.70	86.0	< 0.001	1.14*10^7^	-7.12*10^6^	1.01*10^-23^	
unknown 1	16.48	94.7	< 0.001	6.28*10^5^	-6.12*10^5^	5.72*10^-18^	
unknown 2	20.22	96.5	< 0.001	7.41*10^5^	-7.04*10^5^	5.50*10^-14^	
unknown 3	23.51	94.1	< 0.001	1.93*10^6^	-1.96*10^6^	4.80*10^-48^	

Compounds found in higher amounts in diluted samples and control							
furfural	9.18	82.8	< 0.001	1.03*10^6^	1.34*10^7^	1.49*10^-30^	

Compounds detected in the headspace of the skin microbiota samples, but not present or in significantly higher or lower amounts in the diluted samples and control (exponential regression, P < 0.01). Increase in the abundance of compounds is explained by the parameters in the exponential regression model (A + B*(R**X); X = concentration of bacteria). Numbers in last column refer to studies in which compounds were reported previously. All compounds were identified on the basis of matching spectra (NIST library), and verified by injection of standard (except 2-hydroxy-3-pentanone). ^1^Compounds tested in MM-X traps. RT = retention time. R^2 ^= coefficient of determination.

A significant reduction of furfural was found when microbiota concentrations increased (Table 3, Figure 5; P < 0.001).

### Staphylococcus epidermidis

**Figure 6:**
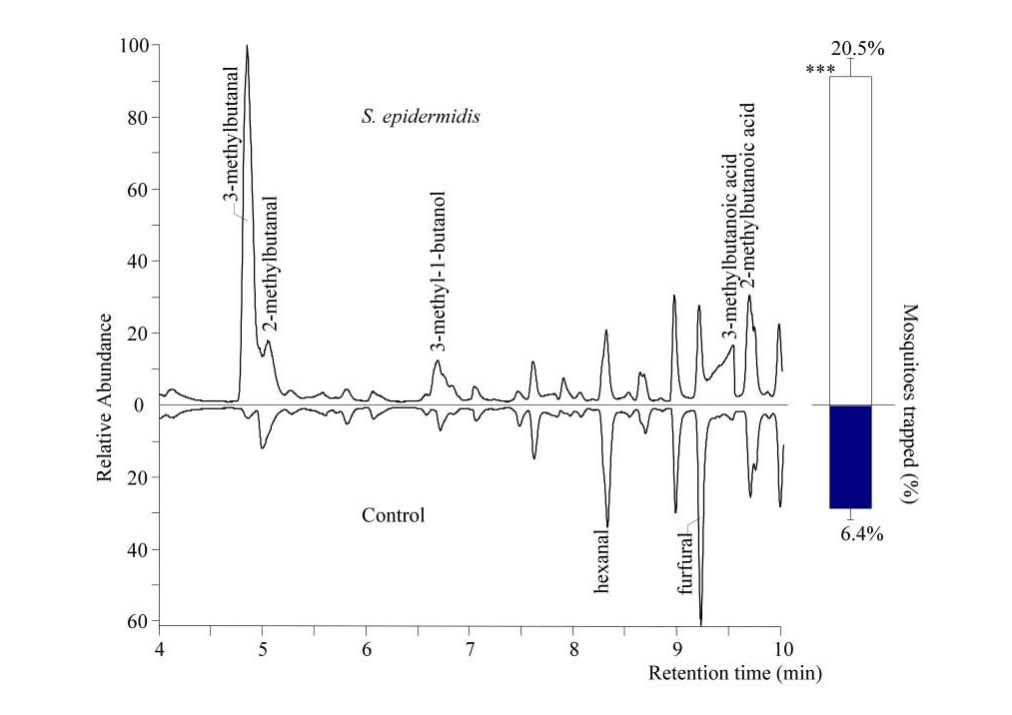
**GC-MS chromatogram of the headspace of *S. epidermidis *and the behavioural response of *An. gambiae***. Part of a GC-MS chromatogram of the headspace of a blood agar plate with *S. epidermidis *and a blood agar plate without skin microbiota indicating the compounds that were present in significantly different amounts (Figure 7). Compound names are indicated when present in significantly higher or lower amount in the treatment compared to the control. Bars represent the average response of released *An. gambiae *in a dual-port olfactometer to both odour sources. Error bars represent standard errors of the mean; ***: χ^2^-test P < 0.001.

**Figure 7:**
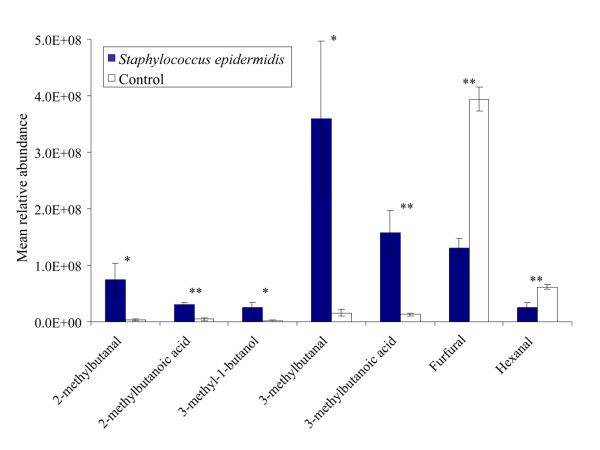
**Mean relative abundance of headspace compounds emitted by *S. epidermidis *on blood agar**. Compounds were present in significantly different amounts in the headspace of blood agar plates with *Staphylococcus epidermidis *than in that of blood agar plates without *S. epidermidis *(Control). Error bars represent standard errors of the mean; **: χ^2^-test P < 0.01; *: χ^2^-test P < 0.05.

Blocks (1.5 × 1.5 × 0.3 cm) of blood agar with a reference strain of *S. epidermidis *caught significantly more *An. gambiae *than sterile blocks of blood agar (Figure 6; χ^2^-test, d.f. = 1, P < 0.001). Headspace analysis revealed five compounds that were present in the headspace of *S. epidermidis *samples, but were absent or present in significantly lower quantities in the control of blocks of sterile agar (Figure 6, 7; ANOVA, d.f. = 1, P < 0.05). These five compounds were also found in the headspace of the microbiota collected from the human foot. Two other compounds, furfural and hexanal, were found in reduced quantities in the headspace of the *S. epidermidis *samples compared to the control (Figure 6 and 7; P < 0.05).

### Experimental room trapping

**Figure 8:**
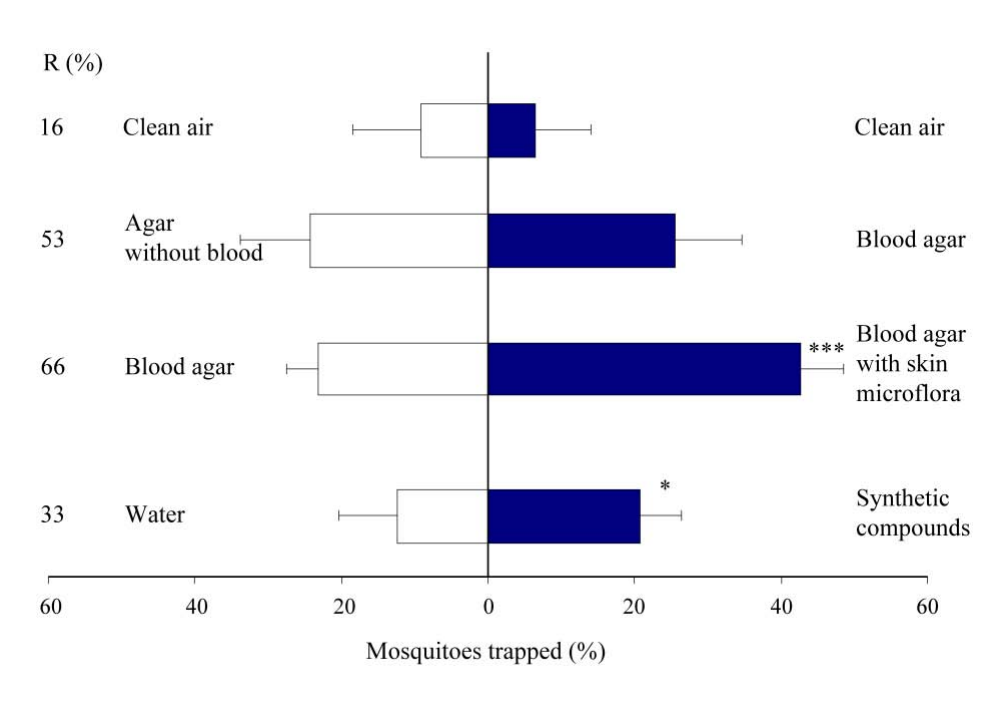
**Mean response of *An. gambiae *in the experimental room trapping experiments**. Error bars represent standard errors of the mean; ***: χ^2^-test P < 0.001; *: χ^2^-test P < 0.05. R = Trap entry response (%).

To test the attractiveness of the volatiles produced by foot microbiota to *An. gambiae *on a larger scale, skin microbiota samples on blood agar (2.63·10^2 ^cfu per cm^2^) were tested against sterile blood agar in a dual-choice test using MM-X traps. A MM-X trap baited with skin microbiota caught significantly more mosquitoes (Figure 8; χ^2^-test, d.f. = 1, P < 0.001) than a MM-X trap baited with sterile blood agar only. The MM-X traps together caught on average 66% of the mosquitoes released.

Sterile blood agar tested in the MM-X setup against sterile agar without blood showed no significant differences in mosquito numbers caught (Figure 8; χ^2^-test, d.f. = 1, P = 0.74). On average, 52.5% of the mosquitoes that left the release cage were caught.

Two unbaited MM-X traps caught equal numbers of mosquitoes showing that the system was symmetrical (Figure 8; χ^2^-test, d.f. = 1, P = 0.24). Together the unbaited traps caught on average 16% of the released mosquitoes.

### Synthetic blend

The MM-X trap containing the ten synthetic odours caught significantly more mosquitoes than the control trap (Figure 8; χ^2^-test, d.f. = 1, P = 0.011). Thirty-three percent of the mosquitoes that left the release cage were caught by the two traps (Figure 8).

## Discussion

Good understanding of olfactory-mediated behaviour is crucial for the development of mosquito control strategies using host-derived semiochemicals [31]. Through the analysis of organic volatiles produced by microbiota isolated from the human skin, compounds were identified that affect the host-seeking behaviour of *An. gambiae*. This finding underlines the important role of microbiology in the elucidation of mosquito-host interactions. Using the compounds identified a synthetic mixture attractive to this mosquito species was developed.

Although blood agar alone was attractive to the mosquitoes, the microbiota mixture used in this study caught significantly more mosquitoes than sterile blood agar, and it is therefore concluded that the volatiles produced by the microbiota themselves are chiefly responsible for the observed effects. The blood agar used in these experiments is a medium rich in organic substances and many of the volatiles identified in the headspace analysis of the blood agar plates incubated with bacteria were also found in the sterile blood agar plates (Figure 5, 6, 7). This could explain why blood agar itself was attractive to the mosquitoes. However, the abundance and composition of volatiles emanated by incubated blood agar plates was much different from those of sterile blood agar plates, causing the mosquito's choice for the former. Volatiles associated with blood can be attractive to mosquitoes [32]. The indoor trapping experiments with agar with and without blood however, showed that blood itself was not a source of attractiveness for the mosquitoes (Figure 8).

The results obtained with skin microbiota are likely representative for humans in general, as the skin microbiota from five men caused attractiveness to *An. gambiae*. To determine the possible correlation between skin microbiota composition and the attractiveness of humans to malaria mosquitoes, the attractiveness of the volunteers needs to be investigated. A research like this would require a higher number of volunteers [3].

Classification of the microbiota in the foot samples used in the first and second olfactometer experiments showed that staphylococci were most abundant and corynebacteria occurred in low numbers only. Micrococci and *Pityrosporum *were not found on the foot of the volunteer, although these are reportedly present on the feet of 30-58% of healthy humans [26,33]. *Staphylococcus *species have been reported to produce 3-methylbutanoic acid [34], a compound that was also detected in the headspace analysis in this study and is associated with foot malodour [35]. Corynebacteria and propionibacteria are capable of catabolizing skin lipids to Long Chain Fatty Acids (LCFAs; C14-C30) and LCFAs to Volatile Fatty Acids (VFAs; C2-C12) [36,37], which were also present in our headspace samples (2-methylbutanoic acid, 3-methylbutanoic acid). Previous studies have shown that aliphatic carboxylic acids play a role in the host-seeking behaviour of *An. gambiae *[11,22,38].

The human odour profile consists of more than 350 compounds [15,16]. Eight of the 14 putative attractive compounds found in the present study have been reported previously from studies on human odour or human sweat [15,16,39-41] (Table 3), which links our results on odour production by *in vitro *cultured skin microbiota to these previous studies.

With the new approach presented here it is possible to identify compounds that affect the host-seeking behaviour of *An. gambiae*. Although there is no information on the correlation between the release rate of the compounds present in the synthetic odour blend and the actual concentration of the odorant chemicals in the headspace of the microbiota, the behavioural response to a blend of 10 compounds that were abundantly present among the bacteria-derived VOCs suggests that this strategy of kairomone identification is an effective means of kairomone discovery. Testing the blend of ten compounds in indoor trapping experiments represents an intermediate research step between laboratory-based olfactometer studies and (semi-)field studies, as the MM-X traps are currently also used in semi-field and field experiments in Africa [42-44].

Although the synthetic blend was more attractive than the control, the trap catches were lower than when skin microbiota or agar alone, were tested. Both quantity and quality of constituents present in synthetic blends are known to have an effect on trapping efficacy [11,22,45,46]. As the release rates of the chemicals from the sachets depended on the volatility of the compounds and the size and thickness of the LDPE sachets [28] (Table 2), one would expect that a stronger positive effect of the blend can be achieved by influencing the release rates of the individual components in the blend by variation of these characteristics. In addition, some compounds that were present in the blend may have had a negative effect on the attractiveness, depending on concentration, and other compounds that may increase the attractiveness of the blend may be missing [47].

Inhibition of the metabolism of certain skin bacteria may reduce a person's attractiveness to malaria mosquitoes. Indeed, some compounds like citral, citronellal and geraniol block foot odour-producing enzymes in bacteria [35,48]. This knowledge can lead to the development of a new class of odour-masking or inhibitory compounds, which can be exploited in the protection from mosquito bites, aiming at compounds that reduce the production of attractive volatiles on the human skin.

Skin microorganisms are known to determine the human odour profile [20] and with the results presented here it is plausible that the composition of the skin microbiota determines an individual's attractiveness to malaria mosquitoes. The discovery that human skin microorganisms mediate malaria mosquito behaviour provides new opportunities for the control of this disease, for example biotechnological approaches using bacteria for mass production of mosquito attractants or modification of the composition of the microbial flora on the human skin to reduce attractiveness.

## Conclusions

Skin microorganisms attract malaria mosquitoes when grown on blood agar in both olfactometer and indoor trapping experiments. A study with five volunteers showed that this effect is probably representative for humans in general. Analysis of the headspace of the skin bacteria in a dilution series resulted in fourteen putative kairomones. A synthetic blend of 10 of these was attractive to *An. gambiae*. This approach to identify semiochemicals could potentially be a novel means of vector-borne disease control through the deployment of semiochemical-baited trapping systems [31,49]. Further knowledge of the effect of skin bacteria on mosquito attraction could support the development of new repellents by blocking skin bacteria or the mass production of skin bacteria as mosquito attractant.

## Competing interests

The authors declare that they have no competing interests.

## Authors' contributions

The initial experimental set-up was developed by NOV, RCS, WT and BGJK. NOV conducted the behavioural experiments and drafted the manuscript. HB conducted the headspace analysis. GS and HJB provided technical advice. RCS, WT and BGJK contributed to drafting the final manuscript. All authors read and approved the final manuscript.
